# Supplementary material for: Homeostatic plasticity in the retina is associated with maintenance of night vision during retinal degenerative disease
Source: eLife. 2020 Sep 22;9:e59422. doi: 10.7554/eLife.59422 (PMC7529457; doi:10.7554/eLife.59422)
Supplement: Transparent reporting form [file elife-59422-transrepform.docx]

***eLife’s* transparent reporting form**

We encourage authors to provide detailed information *within their submission* to facilitate the interpretation and replication of experiments. Authors can upload supporting documentation to indicate the use of appropriate reporting guidelines for health-related research (see [EQUATOR Network](http://www.equator-network.org/%20)), life science research (see the [BioSharing Information Resource](https://biosharing.org/)), or the [ARRIVE guidelines](http://www.plosbiology.org/article/info:doi/10.1371/journal.pbio.1000412) for reporting work involving animal research. Where applicable, authors should refer to any relevant reporting standards documents in this form.

If you have any questions, please consult our Journal Policies and/or contact us: [editorial@elifesciences.org](mailto:editorial@elifesciences.org).

**Sample-size estimation**

- You should state whether an appropriate sample size was computed when the study was being designed
- You should state the statistical method of sample size computation and any required assumptions
- If no explicit power analysis was used, you should describe how you decided what sample (replicate) size (number) to use

Please outline where this information can be found within the submission (e.g., sections or figure legends), or explain why this information doesn’t apply to your submission:

We determined expected (minimum) number of biological replicates required based on SD and mean from our previous experiments with the techniques used in this manuscript (*in vivo* ERG, *ex vivo* ERG, histology, OMR). RNAseq experiment was designed to be sufficiently powered to find differences in gene expression between control and P23H and also potential differences on the effect of P23H mutation between sexes. As our experiments did not find mutation-specific sex differences, data from both sexes were pooled.

To keep power calculations simple, we focused on *R_max_* in ERG experiments and contrast sensitivity (CS) at a single luminance, where CS was about half of the CS_max_, for the OMR experiments. Sample size calculations were based on t-test with alpha = 0.05 and power = 0.8. Depending on expected differences between control and P23H group, we calculate the sample size required to detect either 50% (ERG a-wave), 30% (ERG bipolar cell component, OMR) or 20% (histology) difference in the means for control and P23H mice.

**Replicates**

- You should report how often each experiment was performed
- You should include a definition of biological versus technical replication
- The data obtained should be provided and sufficient information should be provided to indicate the number of independent biological and/or technical replicates
- If you encountered any outliers, you should describe how these were handled
- Criteria for exclusion/inclusion of data should be clearly stated
- High-throughput sequence data should be uploaded before submission, with a private link for reviewers provided (these are available from both GEO and ArrayExpress)

Please outline where this information can be found within the submission (e.g., sections or figure legends), or explain why this information doesn’t apply to your submission:

Information of replicates is given in Figure/Table legends and/or in Materials and Methods. We specify both the number of biological replicates (number of mice) as well as technical replicates (number of retinas/eyes). For behavior experiments, we give number of mice (biological replicate) as well as number of technical replicates (sometimes multiple experiments were performed with the same animal at different days). **All statistical analyses were performed using biological replicates as N.**

No data was excluded unless there was a known technical problem with the experiments, in which case the experiment was terminated, and data not was not analyzed.

Analyzed RNA-seq data comparing transcriptomes of heterozygous P23H mice and their littermate control mice (C57Bl/6N background) at 1 and 3 month of age were uploaded as supporting files with the submission as well as into **GEO database**. Raw data is freely available in the Gene Expression Omnibus (GEO) database (https://www.ncbi.nlm.nih.gov/geo/) with accession numbers GSE152474 (1-month-old samples) and GSE156533 (3-month-old samples)

**Statistical reporting**

- Statistical analysis methods should be described and justified
- Raw data should be presented in figures whenever informative to do so (typically when N per group is less than 10)
- For each experiment, you should identify the statistical tests used, exact values of N, definitions of center, methods of multiple test correction, and dispersion and precision measures (e.g., mean, median, SD, SEM, confidence intervals; and, for the major substantive results, a measure of effect size (e.g., Pearson's r, Cohen's d)
- Report exact p-values wherever possible alongside the summary statistics and 95% confidence intervals. These should be reported for all key questions and not only when the p-value is less than 0.05.

Please outline where this information can be found within the submission (e.g., sections or figure legends), or explain why this information doesn’t apply to your submission:

Details of statistical analysis is presented in Figure/Table legends and in Materials and Methods section.

(For large datasets, or papers with a very large number of statistical tests, you may upload a single table file with tests, Ns, etc., with reference to sections in the manuscript.)

**Group allocation**

- Indicate how samples were allocated into experimental groups (in the case of clinical studies, please specify allocation to treatment method); if randomization was used, please also state if restricted randomization was applied
- Indicate if masking was used during group allocation, data collection and/or data analysis

Please outline where this information can be found within the submission (e.g., sections or figure legends), or explain why this information doesn’t apply to your submission:

N/A. Direction of grating in OMR experiments was masked from experimenter who was deciding whether the mouse was observing clockwise or counterclockwise movement. Mouse genotype was blinded as to the observer in OMR experiments.

**Additional data files (“source data”)**

- We encourage you to upload relevant additional data files, such as numerical data that are represented as a graph in a figure, or as a summary table
- Where provided, these should be in the most useful format, and they can be uploaded as “Source data” files linked to a main figure or table
- Include model definition files including the full list of parameters used
- Include code used for data analysis (e.g., R, MatLab)
- Avoid stating that data files are “available upon request”

Please indicate the figures or tables for which source data files have been provided:

Functions and their parameters are explained in detail in Materials and Methods section.
